# Comparison of Glycated Hemoglobin (HbA1c) Values Estimated by High-Performance Liquid Chromatography and Spectrophotometry: A Pilot Study

**DOI:** 10.7759/cureus.56964

**Published:** 2024-03-26

**Authors:** Avik Ray, Shubham Atal, Swati Sharma, Ananyan Sampath

**Affiliations:** 1 Epidemiology and Public Health, Harvard T.H. Chan School of Public Health, Boston, USA; 2 Pharmacology, All India Institute of Medical Sciences, Bhopal, Bhopal, IND; 3 Pharmacology and Therapeutics, Cactus Communications, Mumbai, IND; 4 Medicine, All India Institute of Medical Sciences, Bhopal, Bhopal, IND

**Keywords:** hplc, chromatography, diabetes mellitus, hba1c, spectrophotometry

## Abstract

Background

Invasive blood sample collection followed by high-performance liquid chromatography (HPLC) based analysis is the gold standard for estimating glycated hemoglobin level or HbA1c currently. Spectrophotometry could be an alternative that holds the potential to be translated into a portable, non-invasive device for glycated hemoglobin level estimation. This study compares HbA1c values obtained from HPLC and spectrophotometry.

Methods

Venous blood samples were collected from both diabetic and non-diabetic participants in a cross-sectional study. The samples were subjected to both HPLC and spectrophotometry-based estimation of HbA1c%. The results obtained were compared, and the relationship between the two estimations were assessed.

Results

About 15 diabetic and non-diabetic individuals participated in the study and 28 samples were included in the final analysis. The Pearson’s correlation coefficient was 0.65 (95% CI, 0.37-0.82), indicating that there was a strong positive association. This was further supported by the findings from linear regression analysis with a p-value of <0.001.

Conclusions

The positive correlation between the HPLC and spectrophotometric values supports the hypothesis that spectrophotometry could be an alternative to conventional HPLC for the measurement of HbA1c. This needs to be further validated through larger, well-powered studies.

## Introduction

Diabetes mellitus is a serious metabolic condition that leads to severe morbidity and affects nearly 420 million individuals worldwide [1]. The management protocols involve pharmacological treatment and lifestyle modifications along with regular glucose monitoring. The most commonly used methods of glucose monitoring have evolved over the decades into enzymatic and non-enzymatic electrochemical glucose sensors which detect blood glucose from a finger prick, commonly. These invasive procedures generally reduce patient compliance as they lead to pain, damage to fingers, and risk of infection and measure only instantaneous serum glucose levels rather than long-term glycemic status. The long-term glycemic control is estimated mainly via the glycated fraction of hemoglobin (glycated hemoglobin or HbA1c) and less commonly with other methods such as serum fructosamine [2], glycated albumin [3-5], 1,5-anhydro-D-glucitol [6,7] and urinary myoinositol [8]. The HbA1c is a fraction of hemoglobin that comprises around 5% of the total hemoglobin in non-diabetic individuals but up to 15% in patients with diabetes [9]. HbA1c is generally estimated by the widely accepted high-performance liquid chromatography (HPLC) method [10,11]. Latex agglutination immunoassay [12], isoelectric focusing of globin chains [13], electrophoresis [14], immunoturbidimetric assays [9], and boronated affinity chromatography have also been used [15,16].

Spectrophotometry is a practical application of Beer-Lambert’s law which has been explored and established for many medical uses such as diagnosis of Thalassemia [17], sickle cell anemia, human papillomavirus infection [18], and many other non-medical purposes. Each molecule in its solution form has its own unique absorption spectrum. At a specific wavelength (λ Max), each molecule absorbs the maximum amount of light, at which all studies are performed for the solution.

A pilot study was conducted to estimate HbA1c using a spectrophotometer and it revealed a good correlation between the values obtained from the spectroscopic method and those obtained from the standard HPLC method. However, it was restricted to the assessment of HbA1c in the range of 4-10.5% only [19]. There has also been a proposal to create an in-vitro optical sensor designed to estimate glycated hemoglobin levels [20]. However, a thorough literature review shows that no such technology for the detection of HbA1c through a spectrophotometer exists currently. Using a spectrophotometric approach may serve to overcome the burden of invasive glycemic monitoring along with providing a measurement of long-standing glycemic status.

The objective of this study was to compare HbA1c values obtained from HPLC and spectrophotometer and explore the relationship between both.

## Materials and methods

**Figure 1:**
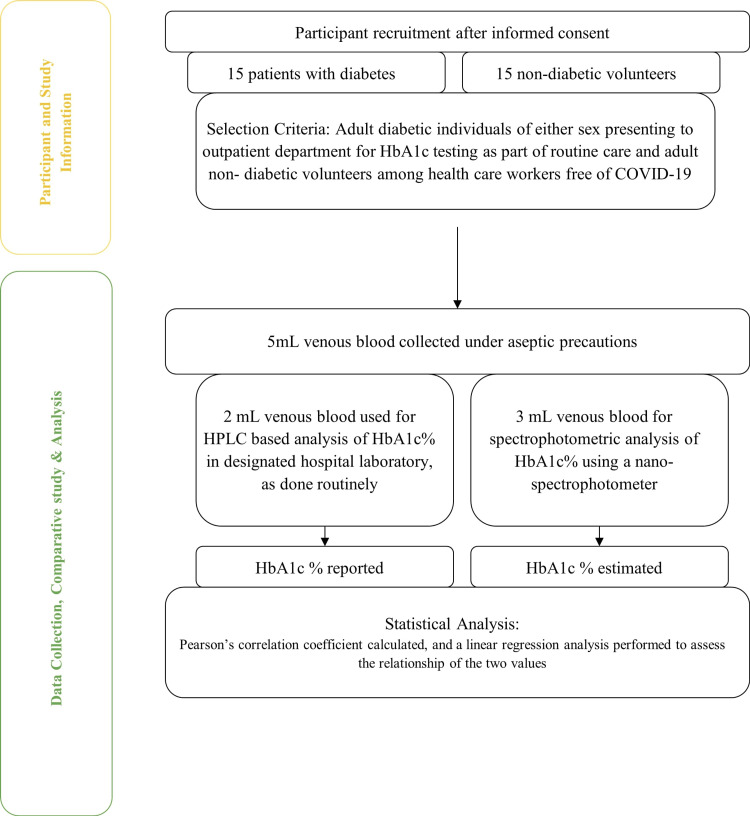
A schematic representation of the study methods HbA1c: Glycated hemoglobin; HPLC: High-performance liquid chromatography

A cross-sectional study was conducted in the Departments of Pharmacology and General Medicine, at All India Institute of Medical Sciences, Bhopal, India wherein adult diabetic patients reporting for consultations in the outpatient department were recruited in the study after taking informed written consent. In parallel, a group of adult non-diabetic individuals (among the healthcare workers) were recruited on a voluntary basis for the study. Both diabetic and non-diabetic participants were included to ensure a wide spectrum of coverage of HbA1c values. A schematic representation of the study methodology is shown in Figure 1.

Venous samples were collected from all participants under aseptic conditions. These were immediately transferred to ethylenediamine tetraacetate containing containers for conducting biochemical analysis.

**Figure 2:**
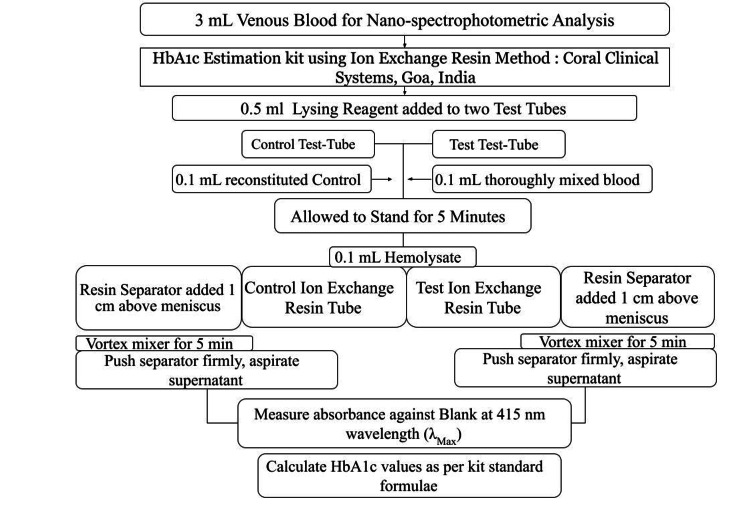
Spectrophotometric analysis of the venous sample for HbA1c analysis HbA1c: Glycated hemoglobin

A portion of the collected blood sample (2 ml) was sent to the accredited laboratory in the institute for standardized HPLC-based routine HbA1c assessment, and the remaining sample (3 ml) was processed through the spectrophotometer (BioTek Instruments Pvt. Ltd., now Agilent India, Bengaluru, India) using a validated kit (Glycosylated Hemoglobin Kit Ion Exchange Resin Method, Coral Clinical Systems, Verna, India) [21] as per the procedure laid out in Figure 2. The wavelength for maximum absorbance (lMax) was found to be 415 nm which was used for the experiment. The investigator analyzing the samples with the nano-spectrophotometer was blinded to the status of the participants in terms of having diabetes or not.

The absorbance values for both test (diabetic samples) and controls (non-diabetic samples) were entered into a Microsoft Excel spreadsheet where the equivalent HbA1c values of the test and comparator samples were calculated as per the kit guidelines, as depicted in Figure 2.

Statistical analysis

Data from the HPLC and spectrophotometric analysis were tabulated onto a Microsoft Excel Spreadsheet. Pearson’s correlation coefficient calculation and linear regression analysis were performed using R version 4.2.2 (R Development Core Team, Vienna, Austria) to assess the association between the two measures.

Ethical considerations

This study was approved by the Institutional Human Ethics Review Board (IHEC) All India Institute of Medical Sciences, Bhopal (IHEC-LOP-2020/IM0306), and GCP guidelines were followed. The participants were provided with informed written consent with provisions to discontinue the study at any point and provided explicit consent to participate in the study and further publish the results without any reference to their personal or identifying information.

## Results

This pilot study comprised 30 participants (15 males and 15 females); two participants were excluded from the final analysis as their samples were deemed unfit. The final analysis was hence performed on 28 participants' data. The participants were a mix of diabetics and non-diabetics (based on HPLC) as defined by American Diabetes Association cut-offs, varying from 5.2% to 13.2% glycation.

The spectroscopic analysis was obtained by kit-based formulae based on the absorbance of the test and standard samples provided in the kit. Each value was individually calculated prior to analysis or comparison between the methods.

The spectroscopy values of the HbA1c did not uniformly parallel with HPLC; the range of HbA1c values as derived via this method was from 4.56% to 13.76%. As this range is larger on both limits in comparison to HPLC, the statistical validity of this method was compared using regression analysis.

Table 1 compares spectrophotometry estimated values of HbA1c vs. HPLC reported values of HbA1c of participants. Table 2 (in the Appendix) depicts the absorbance values used to estimate HbA1c values using spectrophotometry.

**Table 1:** Spectrophotometry predicted values of HbA1c vs HPLC reported values of HbA1c HbA1c: Glycated hemoglobin; HPLC: High-performance liquid chromatography

S. No	Spectrophotometry estimated HbA1c (%) values	HPLC reported HbA1c (%) values
1	9.3	8.6
2	10.2	6.1
3	8.7	5.9
4	8.8	6.8
5	12.8	11.3
6	8.74	5.9
7	9.85	5.3
8	7.03	8.8
9	8.9	9.9
10	7.3	6.4
11	7.02	5.6
12	7.2	7.4
13	4.34	5.5
14	3.9	7.1
15	13.76	13.2
16	4.56	5.2
17	7.42	6.5
18	4.71	5.1
19	7.9	5.7
20	8.44	8.9
21	6.61	6.5
22	5.73	5.8
23	8.9	8.3
24	4.65	6.6
25	8.48	7.6
26	7.36	5.2
27	8.14	6.8
28	8.67	5.8

Table 2 (in the Appendix) shows the individual absorbance values and estimation of HbA1c estimation via the protocol mentioned in Figure 2.

The scatter plot obtained from the participant data consisted of linear form, with a positive association with a moderately strong correlation.

**Figure 3:**
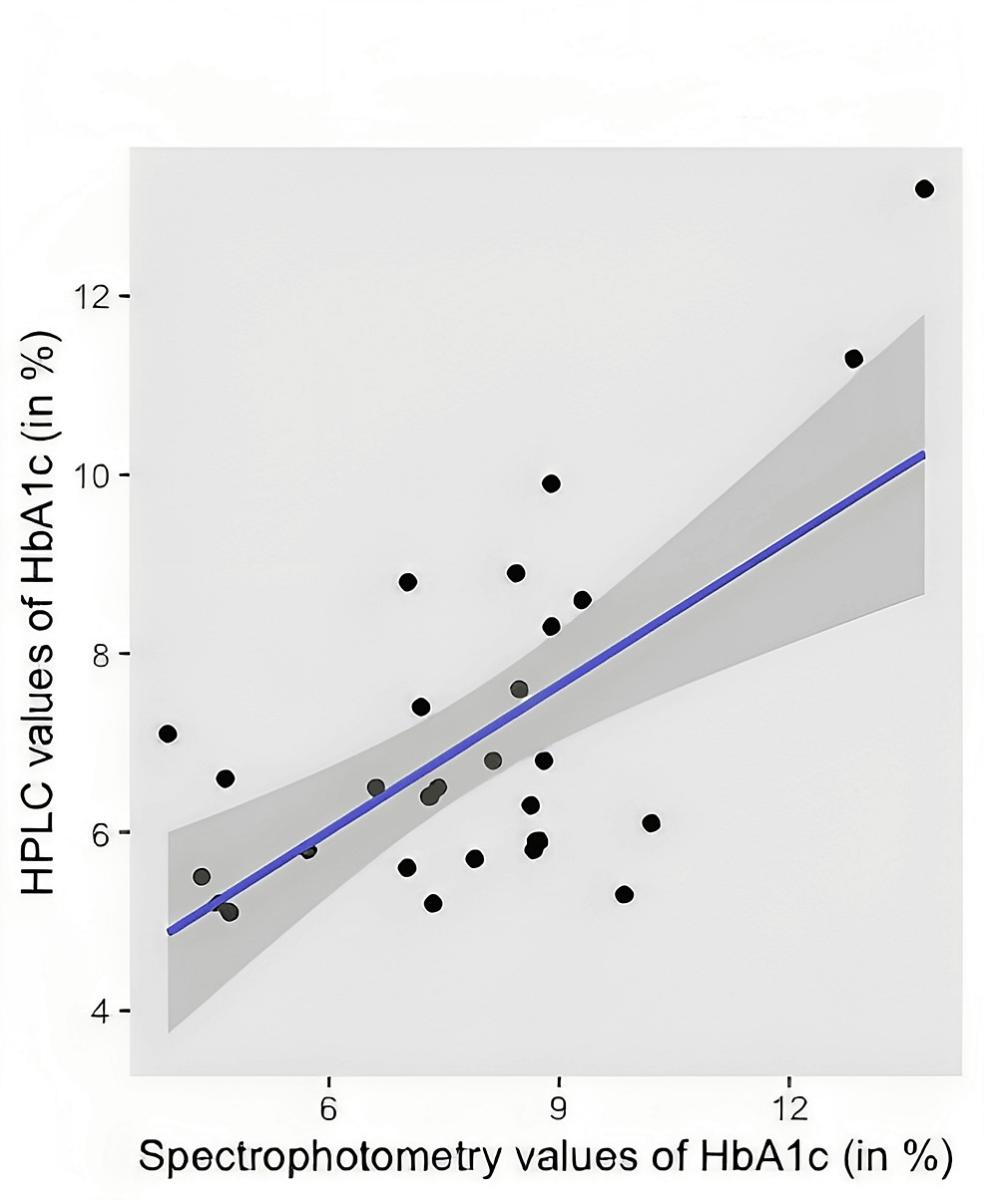
Scatter plot of HPLC vs spectrophotometry HbA1c values HbA1c: Glycated hemoglobin; HPLC: High-performance liquid chromatography

A regression model was designed to predict a relationship, if any, between the two methods, and the plot of best fit was plotted as shown in Figure 3.

The scatter plot demonstrates a linear relationship between the two measures. The Pearson’s correlation coefficient is 0.65 (95% CI, 0.37-0.82) thus indicating a strong positive association over a HPLC-measured HbA1c% range of 5.2-13.2. This is further supported by the findings from the regression analysis with a p-value of <0.001. Spectrophotometry HbA1c% predicted HPLC with an R2 = 0.42, F(1,28) = 19.92, with a p value <0.001, with a β = 76% also significant at p < 0.001. The regression equation came out to be Y = 2.4928 + 0.7628X.

This proves a 64% relationship between the independent (HPLC) and dependent variable (spectrophotometry) in statistically significant limits.

## Discussion

Our findings support the hypothesis that direct absorption spectrophotometry (using a single beam spectrophotometer) of hemolysate could be an alternative to conventional HPLC for the measurement of HbA1c. Absorption spectra obtained using a similar method in an earlier study helped find out the wavelength of maximum absorbance (which came to be around 415 nm as well) for HbA1c along with the absorption peaks of the other derivatives of HbA1c [22]. These findings encourage the thought of developing inexpensive, non-invasive devices based on a similar principle for estimating HbA1c%. The ease of carrying out the test would help overcome patients’ reluctance to get testing done, thus making glycemic monitoring in diabetes simpler.

A reference method from the International Federation of Clinical Chemistry based on electron-spray ionization-mass spectrometry or capillary electrophoresis to estimate levels of HbA(1c) and HbA(0) was developed in Europe [23]. A biosensor for fructose valine working on electrochemical principles for glycosylated hemoglobin detection using magnetic bio-nanoparticles was developed by Chawla and Pundir [24]. They further improvised the method by immobilization of fructose amino acid oxidase on ZnO nanoparticles-polypyrrole film for estimation of HbA1c [25]. Recently, Barman et al. reported a sensitive and specific method to detect the level of HbA1c based on Raman spectrophotometry [26]. Due to its high chemical specificity, Raman spectrophotometry has been considered for the non-invasive screening and diagnosis of diseases. However, in this technique, <1/1,000,000 photons undergo spontaneous scattering. This weakness in the Raman scattered light requires a high-efficiency collection of the light for proper analysis of biological alterations. Berger used laser technology for glucose monitoring but this requires high-end instruments which are very expensive [27].

All these methods had some disadvantages or the other. Many of the mentioned chromatographic methods use two buffers, the first elutes the unbound material from the resin so that it does not lead to desorption of the bound material. The second buffer, which is used at a different pH or ionic strength, is needed to elute the bound material. Various factors such as temperature, pH, and ionic strength are required for the chemical reactions and processes. Additionally, the steps are non-automated or semi-automated and require several different processes, thus increasing the chances of errors.

Given the pilot nature of our study, a direct cost comparison or an economic benefit of either method was not possible as exact estimates or recurring and non-recurring costs were not comparable for both methods. The setting up cost of either method varies significantly, as well as the opportunity cost of the turn-out time for each method. Yet, scaling up newer methods with shorter turn-over times is an essential step in developing faster, more efficient, and point-of-care-abled testing methods for HbA1c, which at the moment is a limitation for the traditional HPLC method.

Our study was not free of these limitations either. We employed a small sample size owing to its hypothesis-generating nature. Further, we could not adjust for potential confounders such as chemical parameters, patient attributes like age, BMI, and underlying comorbid diseases.

Thus, the results so derived require further validation with larger samples and adjusted analyses. Our study lays a strong foundation for further research.

## Conclusions

This pilot study is comprised of a small study population aimed at assessing the technical feasibility of using spectroscopy for measuring HbA1c in individuals. Conventional analysis of HbA1c% has evolved with multiple standardized modalities available today in most tertiary care institutes across India. The most common analysis methods are HPLC-based methods today, which require blood samples. This study was designed to develop and compare an alternative method to HPLC without significant loss in accuracy. A strong correlation of 0.65 was observed between the gold standard HPLC and the spectroscopic method employed in this study, similar to prevailing literature, validating the utility of this method in glycated hemoglobin fraction measurement. Based on the results of this study, spectrophotometry could be developed as an alternative to conventional HPLC to evaluate HbA1c% and warrants further large-scale validation studies.

## Appendices

**Table 2:** Absorbance values of the kit’s control and test samples along with the associated HbA1c values measured by spectrophotometry *Test samples: Participant HbA1c measured by spectroscopy; Control: Kit reagent; HbA1c: Glycated hemoglobin

S. No	Ratio Control* R_c_	Ratio Test* R_T_	R_T/_R_c_	Spectrophotometry estimated HbA1c (%) values
1	0.776	0.675	0.870	9.3
2	0.776	0.682	0.880	10.2
3	0.776	0.9950	1.284	8.7
4	0.776	0.678	0.874	8.8
5	0.776	0.764	0.985	12.8
6	0.776	0.546	0.703	8.74
7	0.776	0.695	0.890	9.85
8	0.776	0.567	0.731	7.03
9	0.776	0.545	0.702	8.9
10	0.776	0.566	0.729	7.3
11	1.805	0.784	0.434	7.02
12	1.805	0.703	0.389	7.2
13	1.805	2.484	1.376	4.34
14	1.805	0.824	0.456	3.9
15	1.805	1.339	0.742	13.76
16	0.983	0.463	0.471	4.56
17	0.983	0.777	0.790	7.42
18	0.983	0.83	0.844	4.71
19	0.983	0.65	0.661	7.9
20	0.983	0.564	0.573	8.44
21	0.972	0.865	0.890	6.61
22	0.972	0.452	0.465	5.73
23	0.972	0.824	0.848	8.9
24	0.972	0.715	0.736	4.65
25	0.972	0.791	0.814	8.48
26	1.072	0.929	0.867	7.36
27	1.072	0.784	0.732	8.14
28	1.702	0.93	0.868	8.67
